# Degree and Rate of Growth Discordance in Dichorionic Twins Conceived by In Vitro Fertilization

**DOI:** 10.1155/2014/543728

**Published:** 2014-07-07

**Authors:** Amira S. Egic, Donka V. Mojovic, Zagorka M. Milovanovic, Aleksandar B. Jurisic, Ljubomir P. Srbinovic, Suzana P. Krsmanovic, Natasa T. Karadzov-Orlic

**Affiliations:** High-Risk Pregnancy Department, Obstetric and Gynecology Clinic “Narodni Front”, University of Belgrade, Street Kraljice Natalije 62, 11000 Belgrade, Serbia

## Abstract

*Objective.* Our objective was to estimate degree and rate of discordant growth and its impact on perinatal outcome in dichorionic twin pregnancies conceived by in vitro fertilization (IVF) compared to those conceived spontaneously. *Study Design.* Growth discordance was defined as 90th percentiles for the study population. Adverse perinatal outcome was defined as 5-minute Apgar score <7 and/or admission to neonatal intensive care unit. *Results.* In the total study population of dichorionic twins (176 conceived by IVF and 215 spontaneously), 30% discordant growth represented the 90th percentile. After adjusting for gestational age, discordant twins conceived by IVF or spontaneously were at higher risk for adverse perinatal outcome (hazard ratio 4.4; 95% CI 2.4–8.3, *P* < 0.0001; hazard ratio 2.5; 95% CI 1.5–4.4, *P* = 0.001, resp.). Similar rates of 5-minute Apgar score <7, admission to neonatal intensive care unit, and delivery <34 weeks were found between discordant twins conceived by IVF and those conceived spontaneously. *Conclusion.* Dichorionic twins conceived by IVF are at similar risk for the rate and degree of discordant growth and adverse perinatal outcome compared to dichorionic twins conceived spontaneously.

## 1. Introduction

Twin gestations are at higher risk for fetal growth restriction and stillbirth compared to singleton gestations [1]. The steep increase in the number of in vitro fertilization (IVF) procedures results in a higher prevalence of multiple pregnancies. Twins conceived by IVF or conceived spontaneously often do not grow at the same rate, and the reported incidence of discordant growth in both pregnancies varies from 10 to 30% [2, 3]. This difference is largely attributed to the use of different definitions of discordance in attempt to increase detection rate of discordant twins who are at higher risk for adverse perinatal outcome (15–30%). Discordant growth is influenced by gestational age at delivery, actual birth weight, gender discordance, chorionicity, growth restriction, twin-to-twin transfusion syndrome, and birth order [3]. In monochorionic twins, who were excluded from this analysis, birth weight discordance is largely attributed to twin-to-twin transfusion syndrome, inequalities in distribution of placental mass between the two fetuses, and abnormalities in cord insertion site [4]. In dichorionic twins, which we analyzed in our study, there might be a difference in genetic growth potential in some cases, but frequently growth discordance is a consequence of placental insufficiency: defective trophoblast invasion or impaired development of uteroplacental circulation [5]. Although the higher risk for defective trophoblast invasion or impaired uteroplacental development has not been reported in pregnancies conceived by IVF, there are greater maternal age, nulliparity, and pregnancy-induced hypertension which may lead to greater degree of discordant growth [3]. Higher rate of discordance (≥30%) for assisted conception has been reported recently [3]. Birth weight discordance is a pathological entity, which may lead to adverse neonatal outcomes such as stillbirth, perinatal morbidity, and preterm delivery [6]. Independently of gestational age at delivery, twins with significant birth weight discordance have poorer perinatal outcomes [6, 7].

Our objective was to determine the rate and degree of growth discordance in pregnancy with dichorionic twins conceived by IVF compared to those conceived spontaneously and to assess association of the growth discordance with adverse perinatal outcome.

## 2. Materials and Methods

This was a retrospective cohort study of all consecutive patients with a twin gestation who underwent routine second-trimester (15–22 weeks) ultrasound for anatomic survey pregnancy control and gave birth from January 2009 to December 2012 in the tertiary medical center, University Clinic “Narodni Front” in Belgrade, Serbia. In this period of time, we analyzed pregnancies with dichorionic twins after exclusion from the analysis: monochorionic twin pregnancies, monoamniotic twins, intrauterine fetal demise of one twin during second trimester, twins with structural anomalies, pregnancies resulting in selective reduction, and pregnancies with incomplete outcome information.

Gestational age was determined by last menstrual period if known and concordant with ultrasound finding (within 7 days of first-trimester ultrasound or 14 days of second-trimester ultrasound) or by the earliest ultrasound when the last menstrual period was unknown. In the pregnancies conceived spontaneously, the gestational age was established by ultrasound examination of the fetal crown-rump length at 11–14 weeks' gestation.

Chorionicity was determined at the earliest ultrasound examination available. First-trimester diagnosis of chorionicity was based on the number of gestational sacs, amnions, and yolk sacs present or the presence of a lambda sign [6]. Second-trimester determination of chorionicity was based on gender discordance, presence of two placental masses, and characteristics of the intertwin dividing membrane (twin peak sign, thickness of membrane) [8].

Birth weight discordance was calculated as the difference between birth weights divided by the birth weight of the larger twin [(larger birth weight − smaller birth weight)]/larger birth weight × 100%)] [9]. Degree of discordance was defined as median value of discordance in spontaneously conceived twins and those conceived by IVF. Degree of discordance was assessed within the subgroups of discordant twins conceived spontaneously or by IVF. All placentae underwent detailed pathologic examination according to a standardized protocol that included final determination of chorionicity. Following enrollment, all pregnancies underwent intensive sonographic surveillance with regular assessment of biometric parameters and also multivessel Doppler studies. Gestational hypertension was defined as blood pressure ≥ 140/90 mmHg measured on two or more occasions at least six hours apart with the patient at rest. Gestational diabetes was defined as carbohydrate intolerance of variable severity with onset or first recognition during pregnancy. Delivery outcome data were collected including mode of delivery, gestational age, birth weight, obstetric complications such as gestational hypertension, gestational diabetes, mode of delivery, birth weight discordance, fetal demise, Apgar score at 5 min, and admission to neonatal intensive care unit (NICU). Low 5 min Apgar score was defined as Apgar score < 7. Adverse perinatal outcome was defined as 5 min Apgar score < 7 and/or admission to NICU (composite end point). Pregnancy outcomes of discordant twin pairs were compared between the two groups conceived by IVF and conceived spontaneously.

Discordance values for the 90th percentile for the study population were determined. The 90th percentile was used in this analysis because it was the lowest percentile associated with adverse perinatal outcome and was frequently used to define normal in a distribution [10].

The study has been approved by Institutional Ethical Committee.

### 2.1. Statistical Analysis

Discordant twins were compared using descriptive and univariate statistics, unpaired Student's* t*-test, or Mann-Whitney* U* test, as appropriate, for continuous variables and Chi square test or Fischer exact test, as appropriate, for dichotomous variables. The level of birth weight discordance was analyzed and illustrated by the larger spatial differences between the Kaplan-Meier curves for dichorionic twins. Cox proportional hazards models were used to analyze morbidity outcomes and the predictive value of birth weight discordance. The Breslow-Day test was used to examine the homogeneity of the hazard ratios between concordant and discordant groups. The Cochran-Mantel-Haenszel test examined the overall association between the outcome and concordance group adjusted for spontaneous grouping. The statistical analysis was performed using statistical software (SPSS Chicago Illinois 17.0).

## 3. Results

**Figure 1:**
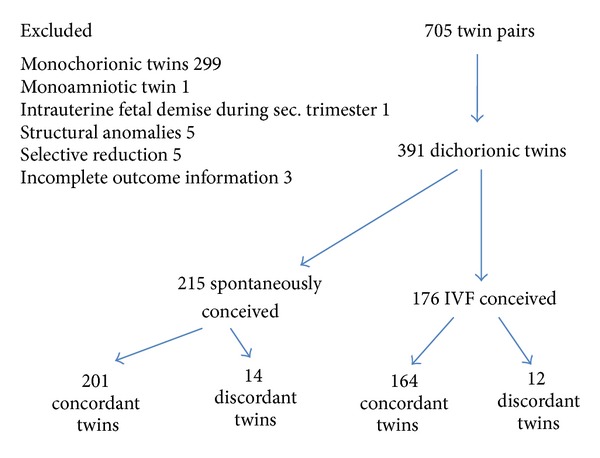
Inclusion of the patients in the analysis.

Out of 27 046 pregnancies 705 were twins. We analyzed 391 pregnancies with dichorionic twins, of which 215 conceived spontaneously and 176 conceived by IVF. In this study, we excluded monochorionic twin pregnancies (299), monoamniotic twins (1), intrauterine fetal demise at the second-trimester ultrasound (1), twins with structural anomalies (5), pregnancies resulting in selective reduction (5), and incomplete outcome information (3) (Figure 1). In the total study population, 30% discordant growth represented the 90th percentile. The incidence of 30% discordance was 6.5% in the group conceived spontaneously and 6.8% in the group conceived by IVF (Table 1). The medians of discordances were similar between the groups (37.8; IQR 22.2 [33.3–55.5] versus 42.4%; IQR 16.7 [35.7–52.4], *P* = 0.58). The mean birth weight of spontaneously conceived twins was 2428.1 ± 564.9 g and that of those conceived by IVF was 2361.2 ± 541.9 g (*P* = 0.32).

In the group of pregnancies conceived spontaneously, twins with ≥30% discordance differed from concordant twins with respect to gestational hypertension (35% versus 9%, *P* = 0.01) and were similar regarding maternal age, nulliparity, and gestational diabetes (Table 1). In the group conceived by IVF, discordant twins were similar to concordant twins in terms of maternal age, gestational hypertension, and diabetes (Table 1). All pregnancies conceived by IVF were nulliparity. Between the groups conceived spontaneously or by IVF, there were no differences in demographic characteristics, except for the maternal age being greater in the IVF group compared to the group with spontaneous conception (33.9 ± 4.87 years versus 30.9 ± 5.02 years, *P* = 0.01).

In pregnancies conceived spontaneously, discordant twins were at increased risk of delivery < 34 weeks (50% versus 16%; RR 0.33, 95% CI 0.18–0.60, *P* = 0.0057) but were not at increased risk of delivery < 28 weeks (0% versus 2%; RR, 0.67; 95% CI, 0.04–11.64) compared to concordant twins (Table 2). In this group, discordant twins were also at higher risk for 5-minute Apgar < 7 (50% versus 19%; RR 0.38; 95% CI 0.21–0.69, *P* = 0.015) and NICU admission (57% versus 19%; RR 0.29; 95% CI 0.18–0.47, *P* = 0.003).

In pregnancies conceived by IVF, discordant twins were at increased risk of delivery < 34 weeks (50% versus 14%; RR 0.28, 95% CI 0.14–0.55, *P* = 0.0055) but were not at increased risk of delivery < 28 weeks (8% versus 3%; RR, 0.36; 95% CI, 0.05–2.89, *P* = 0.35) compared to concordant twins (Table 2). The discordant twins were also at higher risk for NICU admission (67% versus 34%; RR 0.50; 95% CI 0.32–0.79, *P* = 0.029) having a trend toward higher risk for 5-minute Apgar score < 7 (58% versus 31%; RR 0.62; 95% CI 0.35–1.10, *P* = 0.11).

Similar rate of delivery < 34 weeks (50% versus 50%), 5-minute Apgar < 7 (50% versus 58%; RR 1.2; CI 0.66–2.08), and NICU admission (57% versus 67%; RR 0.83; CI 0.41–1.68) were found between the discordant twins conceived spontaneously or by IVF (Table 2).

**Figure 2:**
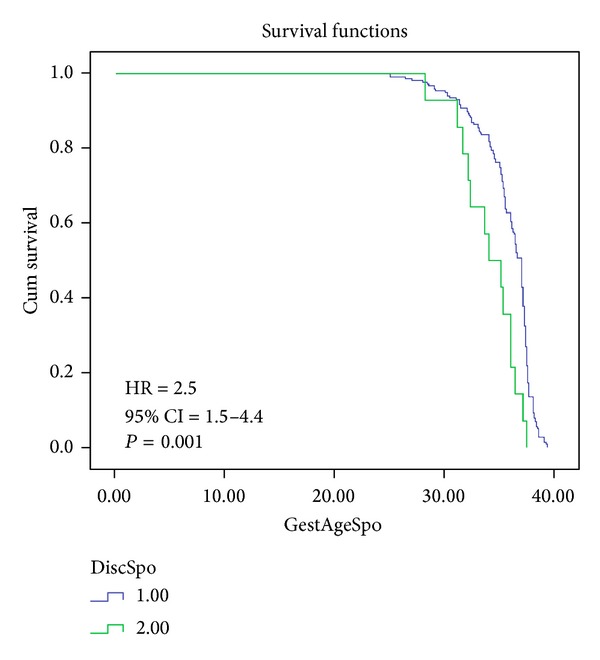
Kaplan-Meier morbidity-free survival curves and birth weight discordance in the group conceived spontaneously. Composite measure of adverse perinatal outcome included 5′ Apgar < 7 and NICU admission. HR: hazard ratio; CI: confidence interval.

**Figure 3:**
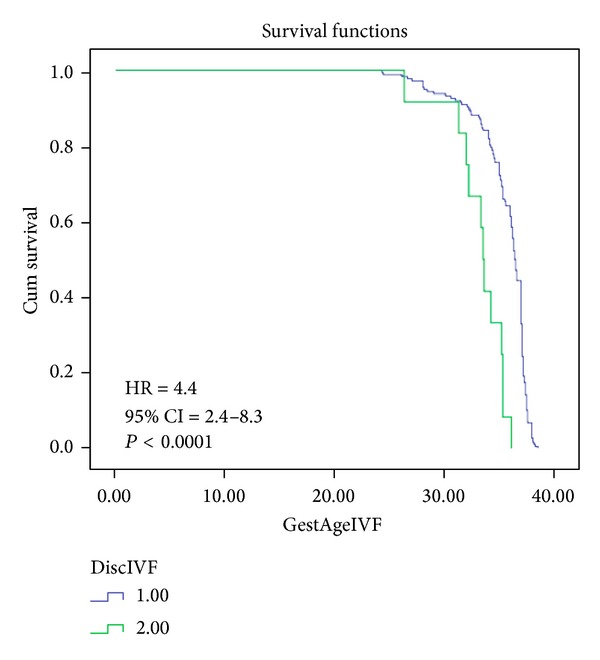
Kaplan-Meier morbidity-free survival curves and birth weight discordance in the group conceived by IVF. Composite measure of adverse perinatal outcome included 5′ Apgar < 7 and NICU admission. HR: hazard ratio; CI: confidence interval.

Kaplan-Meier morbidity-free survival curves were constructed for discordant and concordant twins in pregnancies conceived spontaneously or by IVF (Figures 2 and 3). The risk of poor perinatal outcome (composite end point) was higher among discordant compared to concordant twins conceived spontaneously as illustrated by the larger spatial differences between the Kaplan-Meier curves (Figure 2). After adjusting for gestational age at delivery, discordance of ≥30% for twins conceived spontaneously was associated with higher risk for poor perinatal outcome (hazard ratio 2.5; 95% CI 1.5–4.4, *P* = 0.001). The risk of adverse perinatal outcome (composite end point) was higher in discordant compared to concordant twins conceived by IVF as depicted by greater spatial differences between Kaplan-Meier curves (Figure 3). When adjusted for the gestational age, discordant twins conceived by IVF had a higher risk of adverse perinatal outcome compared to concordant twins (hazard ratio 4.4; 95% CI 2.4–8.3, *P* < 0.0001) (Figure 3). There was no significant difference in the rate of composite end point between the two discordant twins groups, conceived spontaneously or by IVF (78.6% versus 75.0%, *P* = 0.8).

## 4. Discussion

The major findings of our study are as follows: (1) the rate and degree of discordant growth in dichorionic twins conceived by IVF are similar to those in dichorionic twins conceived spontaneously; (2) the rate of adverse perinatal outcome in discordant twin pregnancies conceived by IVF is similar to those conceived spontaneously; (3) the risk of adverse perinatal outcome in dichorionic twin pregnancies was greater in discordant compared to concordant twins in both groups conceived by IVF or spontaneously; (4) preterm delivery (<34 gestational weeks) occurred at similar rate between the two discordant twins groups and at higher rate in discordant compared to concordant twin pregnancies regardless of the mode of conception; (5) adverse perinatal outcome in twin pregnancies with discordant growth is not associated with maternal complications such as gestational diabetes or gestational hypertension, in twins conceived either by IVF or spontaneously.

In this study we analyzed perinatal outcome in two groups of dichorionic twin pregnancies, with assisted and spontaneous conception. Monochorionic twin pregnancies were excluded from the analysis due to their greater perinatal morbidity rate compared to dichorionic twin pregnancies [11]. The two groups of dichorionic twins conceived by IVF or spontaneously were further divided according to birth weight discordance taking the cutoff of 30% which represented the 90th percentile in our total study population. Similar definition for discordant birth weight of 31% representing the 95th percentile was used in the retrospective study of Redman [12]. In that study the authors analyzed 16%, 23%, and 31% of discordant weight finding that weight difference of 31% was associated with the adverse perinatal outcome [12]. The cutoff value for discordance used in another population-based retrospective analysis was 25%, the value based on the data previously reported [13, 14]. In the large multicenter prospective study of 977 monochorionic and dichorionic twin pregnancies, different degrees of discordance were analyzed, 10%, 18%, 20%, and 30%, finding the lowest cutoff of 18% for both monochorionic and dichorionic twins to be associated with twofold increase in risk for adverse perinatal outcome [3]. The risk of adverse perinatal events was threefold greater for dichorionic twin pregnancy with ≥30% discordance compared to discordance <30% [3]. Similarly, in our study the weight difference of ≥30% was associated with 2.5 times increased risk for adverse perinatal outcome in the group of spontaneously conceived twins and 4.4 times increase in risk for adverse perinatal outcome in IVF group. The rates of Apgar score 5′ < 7 and admission to NICU were similar in both groups. It was shown, in our study, that mode of conception had no impact on association between birth weight discordance and adverse perinatal outcome. Currently there is no definite consensus about what degree of discordance in twin pairs should be considered abnormal [15]. It is possible that the cutoff value for significant birth weight discordance could be lower than that reported in retrospective studies [12, 13].

In our study, the rate of discordant growth of 6.8% in IVF group is similar to 6.5% in the group of spontaneously conceived twins. This rate is close to the reported discordance rate of 7.8% in dichorionic twins conceived by IVF [7] and to the rate of 7.0–8.6% of dichorionic twins conceived naturally in other studies using a cutoff value for discordance raging from ≥20% to >25% [7, 13, 16].

Gestational hypertension was more frequently found in pregnancies with discordant compared to concordant growth that conceived spontaneously. This finding is similar to those previously reported that birth weight discordant pregnancies (cutoff value of 25%) had more often maternal hypertension and nulliparity suggesting that maternal factors may contribute to discordant growth in twins [13]. Similar findings recently showed that intertwin birth weight discordance was associated with older maternal age, nulliparity, assisted conception, and pregnancy-induced hypertension [3]. However, in that study, maternal hypertension or preeclamptic toxemia was more frequent in the group with lesser degree of discordance [3]. In the recent study evaluating the perinatal risks associated with growth discordance in dichorionic and monochorionic twin pregnancies these differences in maternal characteristics were not found [17]. In our study in IVF group, there were no differences in maternal characteristics between discordant and concordant growth pregnancies.

Twins with birth weight discordance, with either spontaneous or IVF conception, had higher rate of preterm delivery prior to 34 weeks, but not before 28 weeks. The most frequent cause of preterm delivery in discordant twins was iatrogenic preterm delivery [17]. Discordant dichorionic twins in the recent study with discordant birth weight ≥ 20% were not found to be at increased risk for preterm delivery < 28 weeks, yet there was a trend for delivery < 34 weeks [16].

In conclusion, dichorionic twins conceived by IVF are at similar risk for the rate and degree of discordant growth and adverse perinatal outcome compared to dichorionic twins conceived spontaneously. In dichorionic twin pregnancies conceived either by IVF or spontaneously, birth weight discordance of ≥30% is associated with higher rate of adverse perinatal outcome.

## Figures and Tables

**Table 1:** Maternal demographic data of spontaneous and IVF conceived pregnancies according to the degree of growth discordance.

	Conceived spontaneously			Conceived by IVF		
Demographic characteristics	Concordant 201 (93.5%)	Discordant 14 (6.5%)	*P* value	Concordant 164 (93.2%)	Discordant 12 (6.8%)	*P* value
Maternal age years	30.8 ± 5.0	32.6 ± 5.3	0.2	33.8 ± 4.9	33.9 ± 4.8	0.9
Nulliparity	120 (60%)	9 (64%)	0.9	164 (100%)	12 (100%)	1.0
Gestational Hypertension	19 (9%)	5 (35%)	0.01	17 (10%)	1 (8%)	1.0
Gestational diabetes	12 (6%)	0	1.0	14 (8%)	0	0.6

IVF denotes in vitro fertilization.

**Table 2:** Type of delivery and neonatal outcome stratified by mode of conception.

Conceived spontaneously					Conceived by IVF				Adjusted		
Outcome	Concord 201 (93.5%)	Discord 14 (6.5%)	RR (95% CI)	*P* value	Concord 164 (93.2%)	Discord 12 (6.8%)	RR (95% CI)	*P* value	B-D *P* value	CMH *P* value	RR (95% CI)
PTD < 28 wks	4 (2%)	0	0.67 (0.04–11.64)	1.0	5 (3%)	1 (8%)	0.36 (0.05–2.89)	0.35	0.390	0.672	0.65 (0.09–4.87)
PTD < 34 wks	33 (16%)	7 (50%)	0.33 (0.18–0.60)	0.006	23 (14%)	6 (50%)	0.28 (0.14–0.55)	0.005	0.825	<0.001	0.31 (0.19–0.48)
Cesarean section	111 (55%)	10 (72%)	0.77 (0.54–1.10)	0.28	140 (91%)	12 (100%)	0.91 (0.86–0.95)	0.38	0.311	0.078	0.82 (0.70–0.96)
Apgar score 5′ < 7 (either twin)	38 (19%)	7 (50%)	0.38 (0.21–0.69)	0.015	53 (31%)	7 (58%)	0.62 (0.35–1.10)	0.11	0.645	0.001	0.47 (0.31–0.69)
NICU, either twin	39 (19%)	8 (57%)	0.29 (0.18–0.47)	0.003	55 (34%)	8 (67%)	0.50 (0.32–0.79)	0.029	0.693	<0.001	0.42 (0.30–0.59)
Composite end point	72 (35.8%)	11 (78.6%)	2.5 (1.8–3.5)	0.0001	60 (36.5%)	9 (75.0%)	2.0 (1.3–3.0)	0.0002	0.701	<0.001	2.2 (1.6–3.2)

IVF denotes in vitro fertilization; PTD, preterm delivery; NICU, neonatal intensive care unit; RR, relative risk; CI, confidence interval; B-D, Breslow-Day test; CMH, Cochran-Mantel-Haenszel test.

## References

[1] Spellacy WN, Handler A, Ferre CD (1990). A case-control study of 1253 twin pregnancies from a 1982–1987 perinatal data base. *Obstetrics and Gynecology*.

[2] Blickstein I, Shoham-Schwartz Z, Lancet M, Borenstein R (1987). Characterization of the growth-discordant twin. *Obstetrics and Gynecology*.

[3] Breathnach FM, McAuliffe FM, Geary M (2011). Definition of intertwin birth weight discordance. *Obstetrics and Gynecology*.

[4] Zhao DP, de Villiers SF, Slaghekke F (2013). Prevalence, size, number and localization of vascular anastomoses in monochorionic placentas. *Placenta*.

[5] Kent EM, Breathnach FM, Gillan JE (2012). Placental pathology, birthweight discordance, and growth restriction in twin pregnancy: results of the ESPRiT Study. *The American Journal of Obstetrics and Gynecology*.

[6] Cleary-Goldman J, D’Alton ME (2008). Growth abnormalities and multiple gestations. *Seminars in Perinatology*.

[7] Suzuki S, Miyake H (2010). Perinatal outcomes of elderly primiparous dichorionic twin pregnancies conceived by in vitro fertilization compared with those conceived spontaneously. *Archives of Gynecology and Obstetrics*.

[8] Callen PW (2008). *Ultrasonography in Obstetrics and Gynecology*.

[9] Kingdom JCP, Nevo O, Murphy KE (2005). Discordant growth in twins. *Prenatal Diagnosis*.

[10] Matthews DE, Farewell VT (1996). *Using and Understanding Medical Statistics*.

[11] Oldenburg A, Rode L, Bødker B (2012). Influence of chorionicity on perinatal outcome in a large cohort of Danish twin pregnancies. *Ultrasound in Obstetrics and Gynecology*.

[12] Redman ME, Blackwell SC, Refuerzo JS (2002). The ninety-fifth percentile for growth discordance predicts complications of twin pregnancy. *American Journal of Obstetrics & Gynecology*.

[13] Hartley RS, Hitti J, Emanuel I (2002). Size-discordant twin pairs have higher perinatal mortality rates than nondiscordant pairs. *American Journal of Obstetrics and Gynecology*.

[14] Hollier LM, McIntire DD, Leveno KJ (1999). Outcome of twin pregnancies according to intrapair birth weight differences. *Obstetrics and Gynecology*.

[15] American College of Obstetricians and Gynecologist Committee on Practice Bulletins (2004). Obsterics, Society Form Maternal-Fetal Medicine, ACOG Joint Editorial Committee. ACOG practice bulletin no. 56, multiple gestation: complicated twin, triplet and high-order multifetal pregnancy. *Obstetrics & Gynecology*.

[16] Harper LM, Weis MA, Odibo AO, Roehl KA, Macones GA, Cahill AG (2013). Significance of growth discordance in appropriately grown twins. *The American Journal of Obstetrics and Gynecology*.

[17] Gyamfi-Bannerman C, Fuchs KM, Young OM, Hoffman MK (2011). Nonspontaneous late preterm birth: etiology and outcomes. *American Journal of Obstetrics and Gynecology*.

